# The Voices of Persons Living With Dementia: Exploring Their Information Needs to Live Well

**DOI:** 10.1093/geroni/igaa057.195

**Published:** 2020-12-16

**Authors:** Michelle Kimzey, Ramona Baucham, Chelsea Martin, Carol Howe

**Affiliations:** Texas Christian University, Fort Worth, Texas, United States

## Abstract

There are unique challenges and considerations when receiving the diagnosis of dementia. There are interventions, services, and supports for people with dementia and their care partners, yet they are often unknown, disconnected, and may not be widely available or easily accessible. Health literacy was defined as the degree to which individuals have the capacity to obtain, process, and understand basic health information and services needed to make appropriate health decisions. Using a descriptive qualitative design, the purpose of this study was to describe how persons living with dementia and their care partners obtain, understand, and use information to make health decisions to live well with dementia. The convenience sample consisted of 28 care partners and 15 people living with dementia participating in 6 separate focus groups. To illuminate findings, data was analyzed using a hybrid approach (deductive followed by inductive). Four themes emerged deductively as persons gain health literacy in dementia (access, understand, appraise, and understand). The notable finding is the trend at diagnosis where they first are “seeking the expert” ,and as they move from dependence and gain understanding they are “becoming the expert”, and finally as they apply information they are “acting as the expert” for themselves and others. Engaging them in research not only gave them a voice but more importantly it influenced the health information that will be developed and implemented by them. These findings suggest there is a wealth of knowledge to be gained by persons living with dementia and their care partners.

